# Preoperative maximum standardized uptake value and carbohydrate antigen 19–9 were independent predictors of pathological stages and overall survival in Chinese patients with pancreatic duct adenocarcinoma

**DOI:** 10.1186/s12885-019-5691-4

**Published:** 2019-05-15

**Authors:** Xinjin Gu, Ruiquan Zhou, Chenggang Li, Rong Liu, Zhiming Zhao, Yuanxing Gao, Yong Xu

**Affiliations:** 1 Department of Hepatobiliary and Pancreatic Surgical Oncology, Chinese People’s Liberation Army General Hospital, Beijing, 100853 China; 2 School of Medicine, Nankai University, Tianjin, 300071 China

**Keywords:** Carbohydrate antigen 19–9, Maximum standardized uptake value, Pathological stages, Overall survival, Pancreatic duct adenocarcinoma, Pancreaticoduodenectomy

## Abstract

**Background:**

Purpose of this study was to analyze whether preoperative maximum standardized uptake value (SUVmax) and carbohydrate antigen 19–9 (CA19–9) levels might provide prognostic information in Chinese patients with pancreatic duct adenocarcinoma (PDAC) after pancreaticoduodenectomy (PD).

**Methods:**

Standard PD was performed on 109 patients with PDAC by the same operative team, and all patients received preoperative positron emission tomography/computed tomography examination and blood test.

**Results:**

Patients had a mean age of 59 ± 9.35 years. Females accounted for 38.5%. Mean levels of SUVmax, carcino-embryonic antigen (CEA) and CA19–9 were 5.70 ± 2.76, 3.95 ± 4.16ng/mL and 321.62 ± 780.71kU/L. In univariate Logistic regression analysis, preoperative SUVmax, CEA and CA19–9 levels (*p* < 0.05 for all) rather than other preoperative variables (*p* > 0.05 for all) were significantly related to AJCC stages. Multivariate Logistic regression analysis showed that preoperative SUVmax and CA19–9 levels (*p* < 0.05 for all) rather than other preoperative variables (*p* > 0.05 for all) were significantly associated with AJCC stages. Mean overall survival (OS) was 21 ± 14.50 months. In univariate Cox regression analysis, age, SUVmax, CEA and CA19–9 levels before operation (*p* < 0.05 for all) rather than other preoperative variables (*p* > 0.05 for all) were significantly related to OS. Multivariate Cox regression analysis showed that age, SUVmax and CA19–9 levels before operation (p < 0.05 for all) rather than other preoperative variables (p > 0.05 for all) were significantly associated with OS.

**Conclusions:**

This study demonstrated that preoperative SUVmax and CA19–9 levels independently predicted pathological stages and OS of patients with PDAC after PD. These preoperative variables might have significant prognostic implication in patients with PDAC after PD. Patients with abnormal SUVmax and CA19–9 levels should be paid special attention to in operative strategy and perioperative management.

## Background

Pancreatic duct adenocarcinoma (PDAC) has not only an increasing incidence in many countries, but also the worst prognosis in digestive tract malignancies [1]. In the United States, it is the fourth most common cause of cancer-related mortality and has a 5-year survival rate of less than 5% [1]. As shown in Cancer Statistics in China (2015), its new cases were estimated to be 90.1 thousands and new deaths were estimated to be 79.4 thousands in China. Pancreaticoduodenectomy (PD) is an effective treatment for patients with PDAC and achieves a 5-year survival rate of approximately 15 to 40% [2]. However, regardless of clinically unresectable PDAC (85–90%), these patients with PDAC still have poor prognosis after PD, with a median survival of 11–19 months [3–5]. Early and individualized operative strategy and perioperative management have reduced perioperative mortality of patients with PDAC after PD to < 5% [6, 7]. Preoperative variables would be of great significance to stratify the patients with PDAC and predict the prognosis of patients after PD, and thus promote the early and individualized operative strategy and perioperative management [8].

The most widely applied ^18^F-fluorodeoxyglucose (^18^F-FDG) positron emission tomography/computed tomography (PET/CT)-derived variable designed to measure tracer accumulation is the maximum standardized uptake value (SUVmax), which quantifies the rate of glucose metabolic uptake in tumor cells [9]. Recent studies have considered the SUVmax to be beneficial as prognostic factors in patients with PDAC [10]. However, there are still limited studies regarding the prognostic value of SUVmax in Chinese patients with PDAC after PD. Meanwhile, carbohydrate antigen 19–9 (CA19–9), a sialyated Lewis blood group antigen, is expressed in pancreatic ductal cells [11]. Preoperative CA19–9 levels have been shown to be associated with a significant improvement in postoperative survival in patients with PDAC [12]. However, only a small number of studies have investigated the potential role of preoperative CA19–9 levels as a prognostic variable in Chinese patients with PDAC after PD [13]. Purpose of the current study was to analyze whether preoperative SUVmax and CA19–9 levels might provide meaningful prognostic information in Chinese patients with PDAC after PD.

## Methods

### Study patients

Between May 2010 and May 2016, 121 patients were identified from a prospectively maintained database at Department of Hepatobiliary and Pancreatic Surgical Oncology, Chinese People’s Liberation Army General Hospital, and enrolled into the current study. Inclusion criteria: 1) with pancreatic head cancer; 2) with PDAC confirmed by pathological results; 3) with operable PDAC; and 4) with open PD. There were 12 patients lost during the follow-up and excluded in the current study. Finally, the current study included 109 patients.

### Before operation

Patient demographics, histories and symptoms were obtained before operation. All participants were injected with ^18^F-FDG (5.55 MBq/kg) and scanned by Siemens Biograph 64 high definition PET/CT. Scanned area ranged from the skull base to the upper femur, and obtained image was reconstructed by Ordered Subset Expectation Maximization. Preoperative SUVmax levels were measured from the region of interest in ^18^F-FDG PET/CT image through semi-quantity analysis. Venous blood samples were drawn from all participants, and serum hemoglobin, albumin, total bilirubin, carcino-embryonic antigen (CEA) and CA19–9 were tested using automatic electrochemical luminescence immunoassay method. No patient received chemotherapy or radiotherapy before operation.

### Operative process

All patients underwent standard PD performed by the same operative team at Department of Hepatobiliary and Pancreatic Surgical Oncology, Chinese People’s Liberation Army General Hospital. Supporting tubes were routinely placing in the pancreatic duct for external drainage and removed within one week. The range of lymph node dissection included group 5, 8, 12, 13, 14, and 17 lymph nodes. When the tumor invaded the superior mesenteric vein, partial resection and reconstruction of the superior mesenteric vein were selectively performed according to the intraoperative conditions. However, none of the patients included in the current study had resection and reconstruction of the superior mesenteric artery.

### After operation

All specimens were fixed in the formalin for 24–48 h, and identified by the surgeons and pathologists together. The pathologists then prepared, stained and read the specimen slices. Pathological results were assessed with the 8th Edition of American Joint Committee on Cancer (AJCC) stages [14]. Follow-up examinations were conducted once every 3–4 months during the first 2 years after operation, once every 6 months from 3 to 5 years after operation, and thereafter one time each year. The primary outcome was overall survival (OS). OS was defined as the time from operation to death or follow-up.

### Statistical analyses

Continuous variables were reported with mean and standard deviation. Categorical variables were reported with number and percentage. Univariate and multivariate Logistic regression analyses were applied to analyze whether preoperative variables were significant predictors of pathological stages. Univariate and multivariate Cox regression analyses were applied to analyze whether preoperative variables were significant predictors of OS. Two-sided *P* values < 0.05 were considered as statistical significant. All analyses were conducted with Statistic Package for Social Science software version 20 (SPSS, Chicago, IL, USA).

## Results

### Demographics

As described in Table 1, patients had a mean age of 59 ± 9.35 years. Females accounted for 38.5%. Mean levels of SUVmax, CEA and CA19–9 were 5.70 ± 2.76, 3.95 ± 4.16ng/mL and 321.62 ± 780.71kU/L.

**Table 1:** Description of patients with PDAC after PD

Variables	Description
Age, year	59 ± 9.35
Females (%)	42 (38.5)
BMI	23.25 ± 3.42
Operative histories (%)	8 (7.3)
Diabetes mellitus (%)	18 (16.5)
Hypertension (%)	29 (26.6)
Abdominal pain (%)	32 (29.4)
Back pain (%)	14 (12.8)
Hemoglobin	129.17 ± 14.66
Albumin	39.00 ± 3.82
Total bilirubin	97.75 ± 106.47
CEA	3.95 ± 4.16
CA19–9	321.62 ± 780.71
SUVmax	5.70 ± 2.76
AJCC	
1	57 (52.3)
2–4	52 (47.7)
OS, month	21 ± 14.50

**Abbreviations:** AJCC: American Joint Committee on Cancer; BMI: body mass index; CA19–9: carbohydrate antigen 19–9; CEA: carcino-embryonic antigen; SUVmax: maximum standardized uptake value; OS: overall survival; PDAC: pancreatic duct adenocarcinoma; PD: pancreaticoduodenectomy

### Predictors of AJCC stages

In univariate Logistic regression analysis (Table 2), preoperative SUVmax, CEA and CA19–9 levels (*p* < 0.05 for all) rather than other preoperative variables (*p* > 0.05 for all) were significantly related to AJCC stages. Multivariate Logistic regression analysis (Table 2) showed that preoperative SUVmax and CA19–9 levels (p < 0.05 for all) rather than other preoperative variables (p > 0.05 for all) were significantly associated with AJCC stages.

**Table 2:** Associations between preoperative variables and AJCC stages in univariate and multivariate Logistic analyses

Variables	OR^a^	95CI^a^	*P* value^a^	OR^b^	95CI^b^	*P* value^b^
Age	0.996	0.957–1.037	0.844	0.998	0.941–1.058	0.936
Females	0.994	0.459–2.152	0.988	0.977	0.318–3.055	0.968
BMI	1.057	0.945–1.183	0.329	1.093	0.937–1.275	0.257
Operative histories	0.340	0.065–1.765	0.199	0.259	0.032–2.071	0.203
Diabetes mellitus	0.489	0.169–1.416	0.187	1.033	0.275–3.879	0.962
Hypertension	0.706	0.299–1.667	0.427	0.593	0.184–1.914	0.382
Abdominal pain	1.949	0.843–4.503	0.118	1.390	0.472–4.091	0.550
Back pain	1.545	0.498–4.797	0.451	2.974	0.670–13.198	0.152
Hemoglobin	1.002	0.976–1.028	0.902	1.013	0.966–1.063	0.586
Albumin	0.975	0.883–1.076	0.616	0.914	0.780–1.071	0.266
Total bilirubin	1.001	0.998–1.005	0.532	0.999	0.994–1.005	0.798
CEA	1.259	1.056–1.501	0.010	1.187	0.962–1.465	0.109
CA19–9	1.003	1.001–1.005	0.001	1.003	1.001–1.005	0.009
SUVmax	1.277	1.080–1.510	0.004	1.322	1.073–1.629	0.009

**Abbreviations:** BMI: body mass index; CA19–9: carbohydrate antigen 19–9; CEA: carcino-embryonic antigen; OR: odds ratio; SUVmax: maximum standardized uptake value

**Notes:** ^a^univariate Logistic regression analysis; ^b^multivariate Logistic regression analysis

### Survival analysis

Mean OS was 21 ± 14.50 months. In univariate Cox regression analysis (Table 3), age, SUVmax, CEA and CA19–9 levels before operation (p < 0.05 for all) rather than other preoperative variables (p > 0.05 for all) were significantly related to OS. Multivariate Cox regression analysis (Table 3) showed that age, SUVmax and CA19–9 levels before operation (*p* < 0.05 for all) rather than other preoperative variables (*p* > 0.05 for all) were significantly associated with OS.

**Table 3:** Associations between preoperative variables and OS in univariate and multivariate Cox regression analyses

Variables	OR^a^	95CI^a^	*P* value^a^	OR^b^	95CI^b^	*P* value^b^
Age	1.027	1.002–1.053	0.035	1.034	1.004–1.066	0.028
Females	1.111	0.711–1.738	0.643	0.911	0.544–1.524	0.722
BMI	0.968	0.909–1.032	0.324	0.971	0.903–1.044	0.429
Operative histories	1.455	0.630–3.358	0.380	1.586	0.633–3.976	0.325
Diabetes mellitus	0.669	0.353–1.267	0.217	0.581	0.284–1.189	0.137
Hypertension	1.158	0.716–1.871	0.550	1.120	0.632–1.986	0.697
Abdominal pain	1.265	0.794–2.017	0.323	1.172	0.695–1.977	0.552
Back pain	0.960	0.506–1.823	0.901	1.355	0.658–2.791	0.410
Hemoglobin	0.990	0.976–1.004	0.167	0.995	0.973–1.017	0.636
Albumin	0.983	0.929–1.039	0.545	1.005	0.929–1.088	0.899
Total bilirubin	1.000	0.998–1.002	0.888	1.000	0.998–1.003	0.794
CEA	1.104	1.048–1.163	< 0.001	1.050	0.966–1.141	0.250
CA19–9	1.001	1.000–1.001	< 0.001	1.000	1.000–1.001	0.035
SUVmax	1.129	1.052–1.213	0.001	1.136	1.051–1.228	0.001

**Abbreviations:** BMI: body mass index; CA19–9: carbohydrate antigen 19–9; CEA: carcino-embryonic antigen; OR: odds ratio; OS: overall survival; SUVmax: maximum standardized uptake value

**Notes:** ^a^univariate Cox regression analysis; ^b^multivariate Cox regression analysis

## Discussion

PDAC has a growing trend of incidence and accounts for 1–2% of malignant cancers [1, 2]. Although more patients with PDAC have received the PD, they have not obtained significant improved OS [3–5]. Early and individualized operative strategy and perioperative management have the potential to improve the prognosis of patients with PDAC after PD [6, 7]. It results in a greater focus of preoperative variables predicting the OS [8]. The results from the current study confirmed that preoperative SUVmax and CA19–9 levels were independently prognostic predictors of patients with PDAC after PD.

^18^F-FDG PET/CT is a high-tech imaging method not only accurately indicating anatomical image but also effectively displaying functional metabolism [15]. ^18^F-FDG has become extensively applied as a tracer of PET/CT in clinical imaging of PDAC [16]. As PDAC cells progress and anaerobic glycolysis increases, ^18^F-FDG is taken up more by PDAC cells, converted into 6-P-^18^F-FDG, and stored in PDAC cells. Previous studies have shown that SUVmax plays a significant role in not only the diagnosis, but also staging and prognosis in patients with PDAC [17, 18]. However, conflicting results have been published regarding the prognostic value of SUVmax [19–21]. Moreover, previous studies exploring the value of SUVmax for staging and prognosis have been limited, especially in Chinese patients with PDAC [22]. The current study indicated that SUVmax was a prognostic predictor of OS, suggesting that ^18^F-FDG-PET/CT might serve as an important imaging method that applied to stratify the patient with PDAC and predict the prognosis of patients after PD.

As one cancer marker often applied to screen the PDAC, CA19–9 is the mucoprotein present in pancreatic, biliary, gastric and intestinal epithelium cells [23]. On the one hand, PDAC cells grow up, invade and injure the normal pancreatic and biliary cells, leading to their release of CA19–9; on the other hand, PDAC cells release the CA19–9 by themselves as PDAC grows up. Previous studies have investigated the potential prognostic value of preoperative CA19–9 levels in patients with PDAC [13] However, preoperative variables that may play a predictive role in patients with PDAC are poorly understood in previous studies, especially in Chinese patients. The current study proved that preoperative CA19–9 levels were associated with significant reduced OS and more poor differentiation following PDAC. These findings supported that preoperative CA19–9 levels were not only indicative of tumour burden, but also that preoperative CA19–9 levels might act as a marker of biological aggressiveness [24].

## Conclusions

The current study demonstrated that preoperative SUVmax and CA19–9 levels independently predicted pathological stages and OS of patients with PDAC after PD. These preoperative variables might have significant prognostic implication in patients with PDAC after PD. Patients with abnormal SUVmax and CA19–9 levels should be paid special attention to in operative strategy and perioperative management.

## Abbreviations

^18^F-FDG: ^18^F-fluorodeoxyglucose
CA19–9: Carbohydrate antigen 19–9
CEA: Carcino-embryonic antigen
PD: Pancreaticoduodenectomy
PDAC: Pancreatic duct adenocarcinoma
PET/CT: Positron emission tomography/computed tomography
SUVmax: Maximum standardized uptake value
